# Seven tumor‐associated autoantibodies as a serum biomarker for primary screening of early‐stage non‐small cell lung cancer

**DOI:** 10.1002/jcla.24020

**Published:** 2021-09-23

**Authors:** Ping Chen, Wei Lu, Tingting Chen

**Affiliations:** ^1^ Medical Laboratory The First Affiliated Hospital of Zhejiang Chinese Medical University (Zhejiang Provincial Hospital of Traditional Chinese Medicine) Hangzhou China

**Keywords:** diagnosis, low‐dose spiral computed tomography, non‐small cell lung cancer, tumor‐associated autoantibodies

## Abstract

**Objective:**

The purpose of this study was to analyze the levels of tumor‐associated autoantibodies (TAAbs) in lung diseases and determine their diagnostic efficiency in early‐stage non‐small cell lung cancer (NSCLC).

**Methods:**

We retrospectively analyzed the levels of 7‐TAAbs in 177 newly diagnosed early‐stage NSCLC patients, 202 patients with lung benign diseases and 137 healthy cases. The levels of a panel of 7‐TAAbs, including p53, GAGE7, PGP9.5, CAGE, MAGE A1, SOX2, GBU4‐5, were measured by ELISA.

**Results:**

The serum levels of p53, GAGE7, PGP9.5, CAGE, MAGE A1, SOX2, and GBU4‐5 were not statistically different among NSCLC, benign and healthy groups (*p *> 0.05). The area under the curve (AUC) of 7‐TAAbs was all lower than 0.70. The sensitivity of combined detection was the highest (23.73%), while the specificity was the lowest (88.79%). The positive rates of PGP9.5, SOX2, and combined detection were significantly different among the three groups (*p *< 0.05). Among them, PGP9.5 and combined detection were significantly different between the NSCLC and benign groups (*p *< 0.05), PGP9.5, SOX2 and combined detection were significantly different between the NSCLC and healthy groups (*p *< 0.05).

**Conclusions:**

The diagnostic efficiency of 7‐TAAbs in early‐stage NSCLC was not high, so it cannot be used alone as a screening method for NSCLC.

## Graphical abstract

Comparison of positive rate of 7‐TAAbs among the NSCLC, benign and healthy groups. *P* is the comparison among the NSCLC, benign and healthy groups. *P1* is the comparison between the NSCLC and benign groups. *P2* is the comparison between the NSCLC and healthy groups. *P *< 0.05 was statistically different among the three groups. NSCLC: non‐small cell lung cancer.

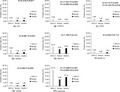

## 1 INTRODUCTION

Lung cancer (LC) has become one of the most common malignances worldwide.^1^ According to histopathological and immunohistochemical staining, LC can be mainly divided into small cell lung cancer (SCLC) and non‐small cell lung cancer (NSCLC). NSCLC is the most common pathological classification, and accounts for more than 80% of LC.^2^ Every year, millions of people are diagnosed as LC and die of it. The 5‐year survival rate of LC varied between 4%–17% due to different pathological stages and types.^3^ LC early diagnosis and immediate treatment can significantly extend patients’ survival, and reduce the mortality by about 20% in high‐risk population under low‐dose spiral computed tomography (LDCT) follow‐up according to the report of the U.S. National Lung Cancer Screening Trial (NLST).^4^ LDCT has high sensitivity and can improve the detection rate of Stage I LC.^5^ However, the false‐positive rate of LDCT was also high, and it reached 96.4% in NLST study.^6^ With the popularization of LDCT, more micro pulmonary nodules have been found by LDCT. In the clinic, LDCT and clinical factors are considered in making a preliminary judgment and a risk rating of pulmonary nodules. However, it is difficult to judge the nature of micro‐nodules accurately based only on imaging and clinical features.^7^ Histopathology, as an invasive mean, is used to identify the properties of these suspicious nodules. According to the guidelines for the management of incidental pulmonary nodules developed by Fleischner Society 2017, CT follow‐up is usually used for some low‐risk nodules due to the unsuitability of invasive methods when considering their very low probability of malignancy.^8^ However, these low‐risk nodules still have a malignant potential, and some patients feel anxious due to this uncertainty. Therefore, it is particularly meaningful to search non‐invasive and high diagnostic efficiency indexes for early‐stage LC screening, not only to avoid delayed diagnosis of malignant LC, but also to reduce the anxiety of patients with benign pulmonary nodules.

Tumor‐associated autoantibodies (TAAbs) are produced by the immune system in response to peptides expressed at the surface of tumor cells or proteins that are released by tumors.^9^ TAAbs have been frequently used in the diagnosis of malignancies, such as colorectal cancer, esophageal cancer, and LC.^10^, ^11^, ^12^ However, scholars have different conclusions on their diagnostic efficiency. Some scholars considered that the incidence of autoantibody reaction against tumor‐associated antigen (TAA) was very low, and these TAAbs should be needed to combine with protein array to improve their predictive value as tumor biomarkers.^13^ At present, TAAbs combined with LDCT have been used in LC screening in some medical institutions. In DU Q study, who chose the same TAAbs panel as ours, he showed that the 7‐TAAbs could distinguish between benign and malignant patients, with the sensitivity of 56.53% and the specificity of 91.60% in the LC diagnosis.^14^ Similarly, our hospital has carried out 7‐TAAbs test for a period of time to assist in LC screening. Unfortunately, we found that the TAAbs panel was not ideal for the initial LC screening. Therefore, we retrospectively analyzed the TAAbs data to explore the diagnostic value in LC, so as to provide a reference for clinical practice.

In our study, we selected a panel of 7‐TAAbs, including p53, GAGE7, PGP9.5, CAGE, MAGE A1, SOX2, and GBU4‐5, for the diagnosis of early‐stage NSCLC. We compared the levels of 7‐TAAbs in NSCLC, benign lung diseases and healthy cases, and explored its diagnostic efficiency in early‐stage NSCLC.

## 2 MATERIALS AND METHODS

### 2.1 Sample information

A total of 177 newly diagnosed early‐stage (stage I and stage II) NSCLC patients in the respiratory department of Zhejiang Province Hospital of Traditional Chinese Medicine were enrolled as NCSLC group from September 2018 to September 2020 (Table 1). All patients were diagnosed according to NCCN guidelines for diagnosis and treatment of primary LC (Version 3.2018).^15^ In addition, 202 patients with benign lung disease who were excluded from LC in the respiratory department were selected as benign group during the same period, and 137 physical examinees without obvious lung abnormalities identified by LDCT, were selected as healthy group. Both benign group and healthy group should exclude malignant diseases. The study was approved by the Zhejiang Province Hospital of Traditional Chinese Medicine.

**TABLE 1:** The clinical features of the NSCLC group

Group		NSCLC group %(*n*)
Pathological classification	Adenocarcinoma	91.53(162)
	Squamous carcinoma	7.91 (14)
	Adenosquamous carcinoma	0.56 (1)
Pathological stage	Stage I	92.09 (163)
	Stage II	7.91 (14)
Typical respiratory symptoms	Cough	12.43 (22)
	Expectoration	8.47 (15)
	Chest pain	1.13 (2)
	Chest tightness	5.65 (10)
Regional lymph node status	N0	97.74 (173)
	N1	2.26 (4)

Inclusion criteria: (1) All cases were 20–90 years old. (2) The NSCLC group was composed of newly diagnosed patients, who had never received surgery, radiotherapy, chemotherapy, or targeted drugs. (3) All NSCLC patients were diagnosed by histopathology, and other tumors were excluded. (4) All NSCLC patients were in stage I or stage II.

Five milliliter venous blood was collected from all cases and centrifuged at 3,000 g/min for 5 min. The serum was separated and used for detection within 8 h. When not immediately tested, the separated serum was stored at −20℃ for detection within 1 week.

The 7‐TAAbs in this study were measured by 7‐TAAbs detection kit (CancerProbe) using the enzyme‐linked immunosorbent analyzer (iMark, Bio‐Rad). Before the experiment, the samples and reagents were redissolved at room temperature for 30 min. The experiment was carried out strictly according to the manufacturer's instructions. In our study, the 7‐TAAbs panel included p53, GAGE 7, PGP 9.5, CAGE, MAGE A1, SOX 2, and GBU 4–5. The reference ranges were as follows, p53: 0.0–13.1 U/ml, GAGE 7: 0.0–14.4 U/ml, PGP 9.5: 0.0–11.1 U/mL, CAGE: 0.0–7.2 U/ml, MAGE A1: 0.0–11.9 U/ml, SOX 2: 0.0–10.3 U/ml, and GBU 4–5: 0.0–7.0 U/ml. If any of the 7‐TAAbs exceeded the reference range, it was considered positive, otherwise it was judged as negative.

### 2.2 Statistical analysis

The SPSS software version 25.0 was used for statistical analysis. The 7‐TAAbs in the NSCLC, benign, and healthy groups were not in accordance to the normal distribution and were expressed as medians (P25, P75). The Kruskal‐Wallis H test was used to compare the levels of the 7‐TAAbs among multiple groups. The Pearson Chi‐squared analysis was used to compare the positive rate. The ROC curve was applied to analyze the diagnostic efficiency of the 7‐TAAbs. A *p*‐value < 0.05 was considered statistically significant.

## 3 RESULTS

### 3.1 Comparative analysis of the levels of 7‐TAAbs among the NSCLC, benign and healthy groups

**FIGURE 1:**
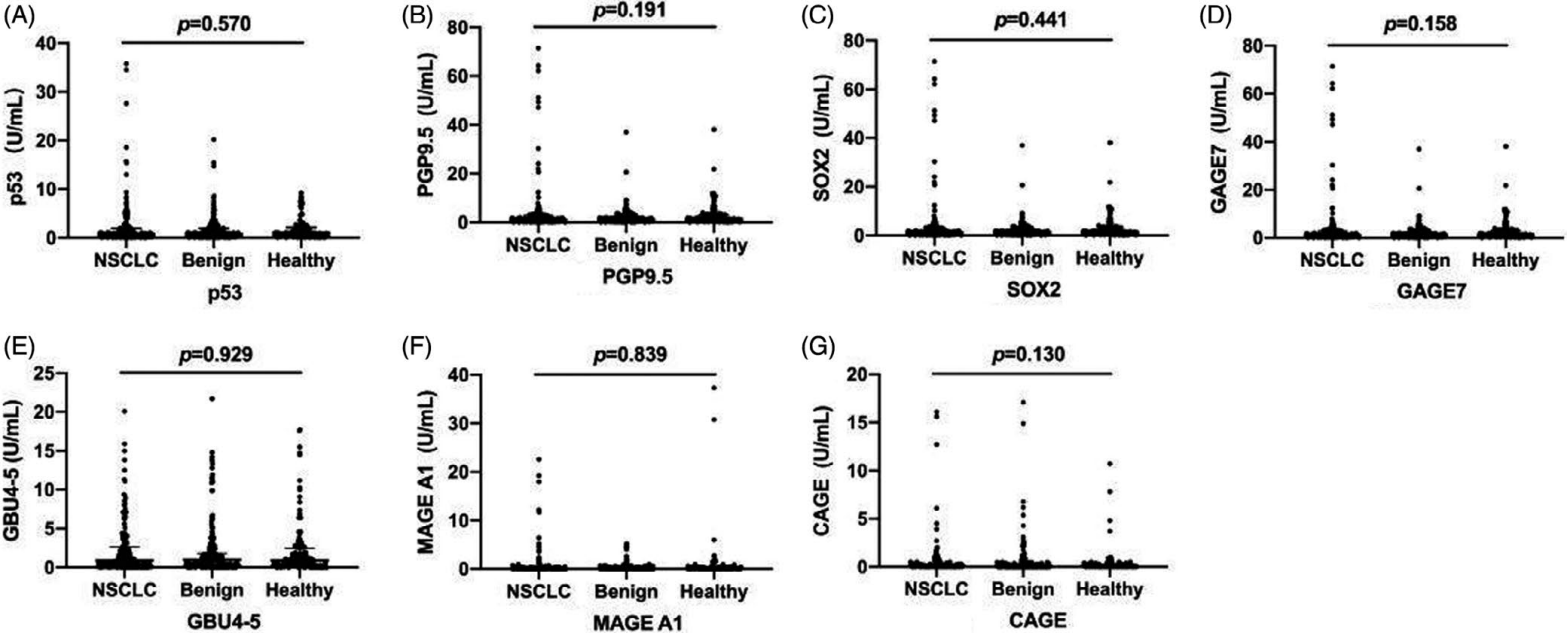
Comparative analysis of 7‐TAAbs levels among NSCLC, benign and healthy groups. (A) Expression Level of p53 in NSCLC, benign and healthy groups; (B) PGP9.5 expression level in NSCLC, benign and healthy groups; (C) SOX2 expression level in NSCLC, benign and healthy groups; (D) GAGE7 expression level in NSCLC, benign and healthy groups; (E) GUB4‐5 expression level in NSCLC, benign and healthy groups; (F) MAGE A1 expression level in NSCLC, benign and healthy groups; (G) CAGE expression level in NSCLC, benign and healthy groups. The serum levels of the 7‐TAAbs were not statistically significant among the three groups (*p *> 0.05)

We compared the serum levels of p53, PGP9.5, SOX2, GAGE7, GBU4‐5, MAGE A1, and CAGE in NSCLC, benign and healthy control groups. The serum levels of the 7‐TAAbs were not statistically significant (*p *> 0.05) among the three groups (Figure 1).

### 3.2 Diagnostic efficiency of 7‐TAAbs in the NSCLC group

**FIGURE 2:**
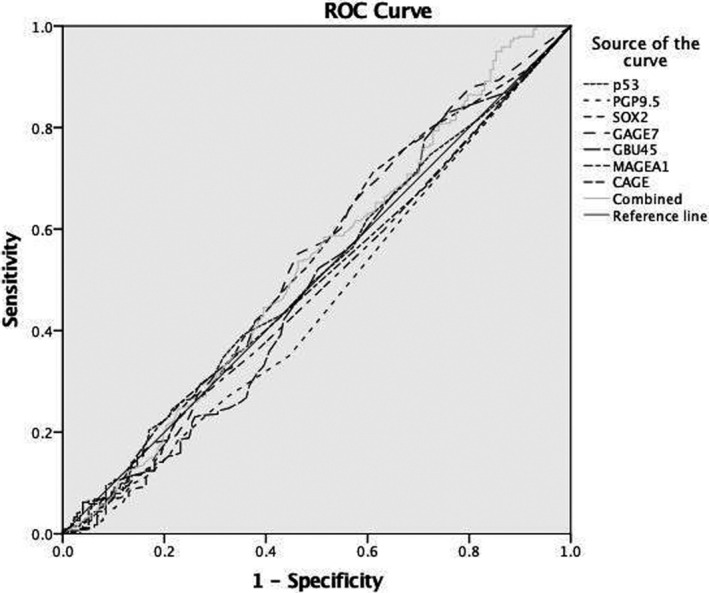
The ROC curve of 7‐TAAbs in NSCLC diagnosis. The NSCLC group was defined as the disease group, and the benign and healthy groups were defined as the control group. The ROC curve of 7‐TAAbs was used to analyze the diagnostic efficiency in NSCLC

The NSCLC group was defined as the disease group, and the benign and healthy groups were defined as the control group. The ROC curve was used to analyze the diagnostic efficiency of 7‐TAAbs in early‐stage NSCLC patients. The area under the ROC curve (AUC) of p53, PGP9.5, SOX2, GAGE7, GUB4‐5, MAGE A1, CAGE, and the combined 7‐TAAbs were 0.508, 0.453, 0.525, 0.532, 0.491, 0.492, 0.482, and 0.527, respectively (Figure 2). The sensitivity of the combined detection was the highest (23.73%), while the specificity was the lowest (88.79%) (Table 2).

**TABLE 2:** The diagnostic efficiency of 7‐TAAbs in NSCLC

	p53	PGP9.5	SOX2	GAGE7	GUB4‐5	MAGE A1	CAGE	Combined
Sen (%)	2.26	3.39	3.95	5.65	10.17	2.26	1.69	23.73
Spe (%)	98.53	100.00	99.12	98.23	92.92	99.41	98.82	88.79
PPV (%)	44.44	100.00	70.00	62.50	42.86	66.67	42.86	52.50
NPV (%)	65.88	66.47	66.40	66.60	66.46	66.08	65.82	69.04

Abbreviations: NPV, negative predictive value; PPV, positive predictive value; Sen, Sensitivity; Spe, Specificity.

### 3.3 The positive rate of 7‐TAAbs in three groups

**FIGURE 3:**
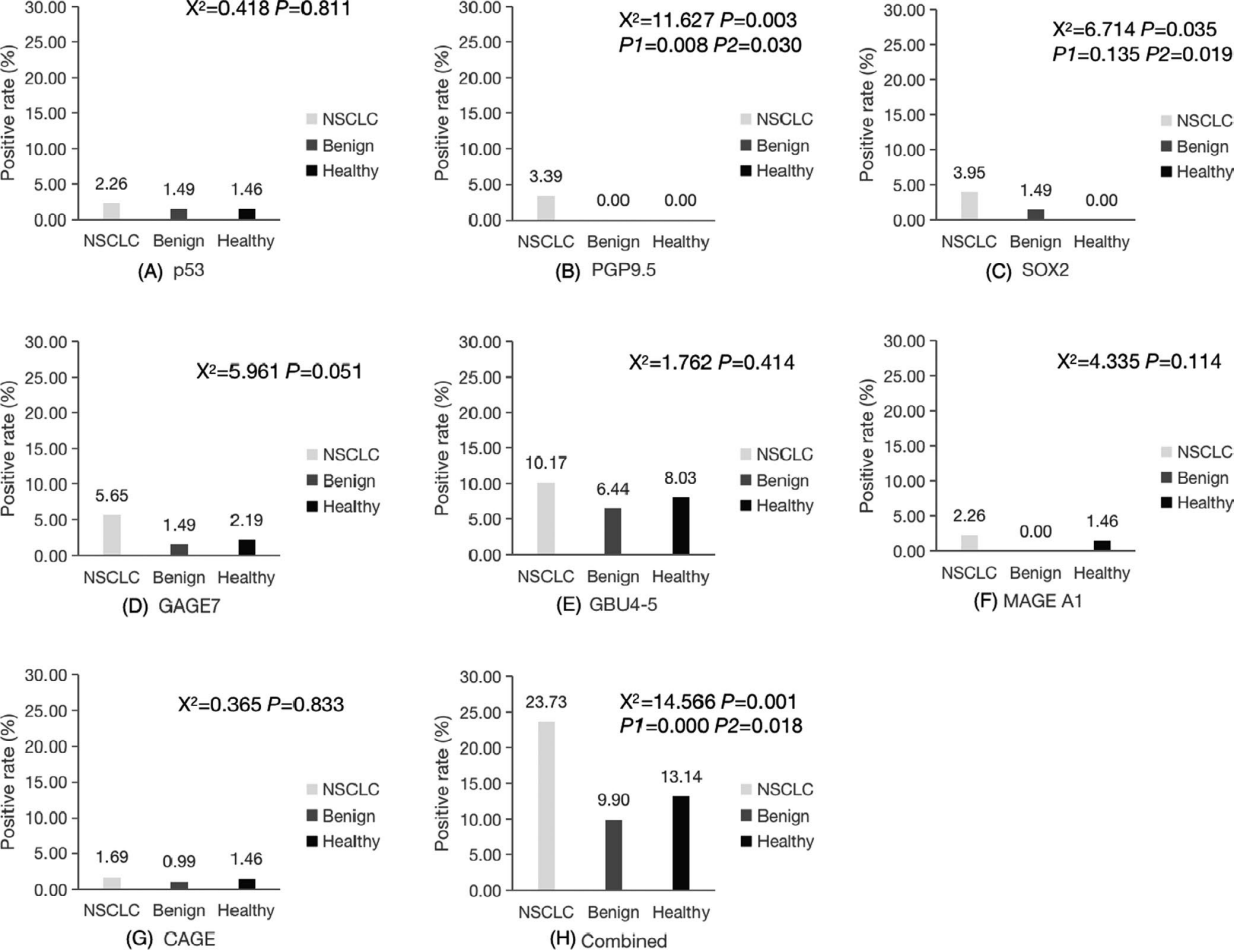
Comparison of positive rate of 7‐TAAbs among the NSCLC, benign and healthy groups. (A) Comparison of the positive rate of p53 among the NSCLC, benign and healthy groups; (B) Comparison of the positive rate of PGP9.5 among the NSCLC, benign and healthy groups; (C) Comparison of the positive rate of SOX2 among the NSCLC, benign and healthy groups; (D) Comparison of the positive rate of GAGE7 among the NSCLC, benign and healthy groups; (E) Comparison of the positive rate of GUB4‐5 among the NSCLC, benign and healthy groups; (F) Comparison of the positive rate of MAGE A1 among the NSCLC, benign and healthy groups; (G) Comparison of the positive rate of CAGE among the NSCLC, benign and healthy groups; (H) Comparison of the positive rate of combined detection among the NSCLC, benign and healthy groups. *p* is the comparison among the NSCLC, benign and healthy groups. *p1* is the comparison between the NSCLC and benign groups. *p2* is the comparison between the NSCLC and healthy groups. *p3* is the comparison between the benign and healthy groups. *p < 0.05 was statistically different among the three groups. NSCLC, non‐small cell lung cancer

We compared the positive rate of a single marker and combined markers in the NSCLC, benign, and healthy groups. The positive rate of combined detection was higher than that of any single detection in the three groups. However, the positive rate was not high in NSCLC group with less than 50.00%. In addition, we compared the positive rate of 7‐TAAbs among the three groups. PGP9.5, SOX2 and combined detection were significantly different among the three groups (*p *< 0.05). PGP9.5 and combined detection were significantly different between the NSCLC and benign groups (*p *< 0.05). PGP9.5, SOX2, and combined detection were significantly different between the NSCLC and healthy groups (*p *< 0.05). There were no statistical differences between the benign and healthy groups (*p *> 0.05), (Figure 3).

## 4 DISCUSSION

Lung cancer screening methods include LDCT, X‐ray, histopathology, and serological detection. With the improvement of health awareness and the popularity of physical examination, LDCT has been extensively used in clinical LC screening because of high sensitivity.^16^ However, LDCT also had high false‐positive rate. Many patients were diagnosed with pulmonary nodules by LDCT, and some people were troubled because of the uncertain nature of these nodules. Histopathology is an invasive examination that is used for the diagnosis of suspected patients. Generally, it is not suitable for all patients who have pulmonary nodules to diagnose. Early detection and treatment can prolong the survival of LC patients. Therefore, it is meaningful to find non‐invasive laboratory indicators that have high diagnostic efficiency. The laboratory method can easily be popularized in the clinic, which is conducive to LC early diagnosis, and reduces the anxiety of some patients diagnosed with pulmonary nodules.

Tumor associated antigens (TAAs) which are highly expressed in the process of tumorigenesis and progression, can induce immune response.^17^ Compared with TAAs, TAAbs have amplification effects and are easily detectable, thus TAAbs can be considered as tumor markers.^13^ In recent years, TAAbs have been used in LC auxiliary diagnosis, however, the conclusion of their diagnostic efficiency was partially consistent.^11^, ^18^ This may be related to the enrolled patients and the selected panel of TAAbs. In the study of LI P, he showed that the levels of some TAAbs increased with the increase of pathological stages.^19^ In our study, the NSCLC group was mainly the adenocarcinoma patients (91.53%) and most of them were in stage I (92.09%), which may lead to the low sensitivity of 7‐TAAbs. With the improvement of health awareness and economic development, physical examination has become a routine, many LC patients were diagnosed in physical examination in our developed areas. In the past, many patients were diagnosed because of clinical symptoms. Compared with the past, LC was detected earlier and the typical symptoms of the respiratory tract in LC patients were not atypical. Cough, hemoptysis, and chest pain were observed in very few patients. Traditional tumor markers, such as carcinoembryonic antigen (CEA), neuron‐specific enolase (NSE), and CYFRA 21–1, also have low sensitivity and specificity in LC early diagnosis.^20^, ^21^ Many researchers focused on finding new serum protein markers with high diagnostic efficiency, such as serum anti‐MDM2 and anti‐c‐Myc.^22^ In our study, there was no significant difference in TAAbs values between NSCLC, benign and healthy groups. The AUC of single and combined 7‐TAAbs were lower than 0.70, indicating that 7‐TAAbs were not effective in LC early diagnosis. Clinically, any one of the 7‐TAAbs that exceeded the reference range, was considered positive. The positive rate of 7‐TAAbs combined detection was 23.73% in NSCLC group, 9.90% in benign group and 13.14% in healthy control group (*p *< 0.05). Although the positive rates of PGP9.5, SOX2 and combined detection were significantly different among the three groups (*p *< 0.05), 7‐TAAbs were not suitable for the initial screening of LC in patients with micro pulmonary nodules, in view of their low sensitivity. In the study by Du Q et al.,^14^ whose 7‐TAAbs panel were consistent with our study, the sensitivity of the combined detection was 56.53%, and the specificity was 91.60%, which were higher than those observed in our study. This difference may be related to the pathological type of our enrolled LC patients. Their LC group included NSCLC and SCLC patients, while our LC group was composed of NSCLC patients. Their LC patients were in different stages, whereas LC cases were mainly in stage I or stage II in our study. In addition to patient factors, the performance of different brands of reagents may also be different. In the Gonzalez Maldonado study,^23^ the sensitivity of the EarlyCDT^®^‐Lung‐a test panel of 7‐TAAbs was only 13.0%, and the specificity was 88.9%. In our study, although the sensitivity was higher than theirs, it was still at a low level, indicating that the panel of 7‐TAAbs was not sensitive enough in the diagnosis of early LC. In Li Y study, he found that the levels of TAAbs was closely related to the progress of the tumor, and TAAbs could be used as an indicator of curative effect monitoring and tumor recurrence in lung adenocarcinoma patients.^24^ Because TAAbs were highly related to cancer progression, and therefore, 7‐TAAbs may be used to monitor the progression and treatment of NSCLC patients. In our study, although the positive rate of combined TAAbs was significantly different among NSCLC, benign and healthy groups (*p *< 0.05), the levels of TAAbs were not significantly different among these three groups. These results demonstrated that 7‐TAAbs could differentiate benign from malignant to some extent, but the diagnostic efficiency was insufficient enough. They showed that 7‐TAAbs may only be used as an auxiliary diagnostic tool, which cannot be used for NSCLC screening alone.

So far, the diagnostic efficiency of tumor markers for LC diagnosis was not high. With the popularity of LDCT, more lung nodules were detected by LDCT, however, only a very low proportion was diagnosed as LC. Our study also has some deficiencies. In the NSCLC group, most patients were in stage I. With the development of medicine and the improvement of health awareness, the pathological stages of many newly diagnosed LC patients were in the early stages. That was the reason why our NSCLC patients were mainly in stage I. However, it is still difficult to diagnose early‐stage LC precisely. Many researchers continue to search for tumor markers of LC, based on molecular biology and immunology. Non‐invasive, easy to be carried out and high diagnostic efficiency projects were urgently needed in clinic, but there is a long way to go.

## 5 CONCLUSIONS

The diagnostic efficiency of 7‐TAAbs in early‐stage NSCLC was not high, so it cannot be used alone as a screening method for LC. In some medical institutions that carried out TAAbs, the results should be carefully analyzed.

## CONFLICT OF INTEREST

No potential conflict of interest was reported by the authors.

## AUTHOR CONTRIBUTIONS

LW collected the clinical data and divided then into different groups according to the inclusion criteria. CP analyzed the clinical data of the NSCLC, lung benign disease and normal control. CTT was a major contributor in writing the manuscript.

## Data Availability

The datasets analyzed during the current study are available from the corresponding author on reasonable request.
